# Evolution of evolvability and phenotypic plasticity in virtual cells

**DOI:** 10.1186/s12862-017-0918-y

**Published:** 2017-02-28

**Authors:** Thomas D. Cuypers, Jacob P. Rutten, Paulien Hogeweg

**Affiliations:** Theoretical Biology Group, Utrecht University, Padualaan 8, Utrecht, 3584 CH The Netherlands

**Keywords:** Evolvability, Regulation, Mutation, Adaptation, Environmental change, Timescales

## Abstract

**Background:**

Changing environmental conditions pose a challenge for the survival of species. To meet this challenge organisms adapt their phenotype by physiological regulation (phenotypic plasticity) or by evolving. Regulatory mechanisms that ensure a constant internal environment in the face of continuous external fluctuations (homeostasis) are ubiquitous and essential for survival. However, more drastic and enduring environmental change, often requires lineages to adapt by mutating. In vitro evolutionary experiments with microbes show that adaptive, large phenotypic changes occur remarkably quickly, requiring only a few mutations. It has been proposed that the high evolvability demonstrated by these microbes, is an evolved property. If both regulation (phenotypic plasticity) and evolvability can evolve as strategies to adapt to change, what are the conditions that favour the emergence of either of these strategy? Does evolution of one strategy hinder or facilitate evolution of the other strategy?

**Results:**

Here we investigate this with computational evolutionary modelling in populations of Virtual Cells. During a preparatory evolutionary phase, Virtual Cells evolved homeostasis regulation for internal metabolite concentrations in a fluctuating environment. The resulting wild-type Virtual Cell strains (WT-VCS) were then exposed to periodic, drastic environmental changes, while maintaining selection on homeostasis regulation. In different sets of simulations the nature and frequencies of environmental change were varied. Pre-evolved WT-VCS were highly evolvable, showing rapid evolutionary adaptation after novel environmental change. Moreover, continued low frequency changes resulted in evolutionary restructuring of the genome that enables even faster adaptation with very few mutations. In contrast, when change frequency is high, lineages evolve phenotypic plasticity that allows them to be fit in different environments without mutations. Yet, evolving phenotypic plasticity is a comparatively slow process. Under intermediate change frequencies, both strategies occur.

**Conclusions:**

We conclude that evolving a homeostasis mechanisms predisposes lineage to be evolvable to novel environmental conditions. Moreover, after continued evolution, evolvability can be a viable alternative with comparable fitness to regulated phenotypic plasticity in all but the most rapidly changing environments.

**Electronic supplementary material:**

The online version of this article (doi:10.1186/s12862-017-0918-y) contains supplementary material, which is available to authorized users.

## Background

Many of Earth’s environments are highly dynamic and unpredictable. Nevertheless, life has mostly found ways to cope with the continuous and the sporadic changes. At the heart of the ability to survive in a range of environmental conditions are regulatory mechanisms that ensure maintenance of internal homeostasis [1–3], which relies on accurate sensing of internal metabolite levels [3, 4]. At the other end of the spectrum of environmental variation lie sporadic, potentially drastic changes that challenge the abilities of organisms to adapt [5–7]. Successful lineages have responded to such events with evolutionary adaptations including lineage specific gene expansions and whole genome duplication. Interestingly, recent experimental evolution studies have shown that evolutionary adaptation can be a remarkably rapid response [8–14] and that surprisingly few mutations are needed to achieve significant fitness gains [9, 13–17]. It has been suggested that the ability to drastically change the phenotype with few mutations, which we shall refer to as evolvability, is itself an evolved property [18–20] (see also Table 1). This leads to the question of how the evolution of evolvability and the evolution of regulated phenotypic plasticity interrelate.

**Table 1:** Definitions

Homeostasis	Homeostasis regulation is the ability to maintain constant levels of some internal molecules while external conditions change continuously, through physiological regulation. In our simulations we require individuals to keep internal concentrations of a resource and energy molecule at a fixed target level, while the concentration of the external resource fluctuates. Although regulatory structure required to maintain homeostasis is free to evolve, it crucially depends on the ability of transcription factors to act as sensors of ligand concentration, allowing the differential regulation of downstream genes.
Phenotypic plasticity	Phenotypic plasticity is the ability of an organism to change its phenotype through regulation. Although typically used to indicate that an organisms can have different morphologies or other external phenotypic traits under varying external conditions, here we mean that an expression pattern of genes can change in a functional way, depending on the external conditions, to allow an individual to retain fitness in different environments.
Evolvability	Different definitions of evolvability have been given in the literature. At a minimum, evolvability is the ability of a genetic system to generate adaptive mutational variation. This ability is influenced by intrinsic properties, such as the mutation rate, the organization of the genotype to phenotype mapping and the different types of mutation that occur. Here we use the term evolvability to indicate the ability to rapidly adapt to environmental change through a small number of mutations. We study whether and how lineages become more evolvable toward recurring shifts in environmental conditions (adapt faster and need less mutations to regain fitness), by evolution of the genotype to phenotype mapping. We refer to this as evolution of evolvability.

Although evolutionary adaptation has traditionally been considered a slow process compared to physiological adaptation the results of aforementioned studies suggest that evolutionary adaptation and phenotypic plasticity can be alternative viable strategies when organisms face repeated changes in environmental conditions. Under which circumstances would one or the other strategy be more likely to evolve? Are there evolutionary paths that lead from one strategy to the other? Here we studied the interplay between these two strategies by performing in silico evolutionary experiments using a Virtual Cell (VC) model [21]. This model was previously used to study a generic pattern of rapid genome inflation and gradual streamlining during long term genome evolution [22] as well as adaptive and neutral effects of whole genome duplications following environmental change [23]. Here, we used wild-type Virtual Cell strains (WT-VCS) that have been pre-evolved for internal homeostasis. Homeostasis was evolved by selecting for the maintenance of constant internal concentrations of a resource and an energy carrier when external resource concentrations fluctuate continuously. We then let pre-evolved WT-VCS adapt to periodic environmental changes with different frequencies, while retaining the selection for internal homeostasis.

We find that when the frequency of changes is low, populations evolve higher evolvability provided that they retain their capacity to regulate resource homeostasis. The evolution of evolvability is evident from a decrease in the number of generations that lineages remain in an unfit state, after environmental change, as well as an increase in the frequency of mutations with large positive fitness effects relative to the alternative environment [18, 19]. When the frequency of environmental change increases populations in addition evolve a distinct phenotypic plasticity strategy. Regulated phenotypic plasticity allows these populations to retain fitness during a drastic change in environmental conditions, without adaptive mutations. However, the evolution of additional gene regulation that is needed for phenotypic plasticity takes place on a much longer time scale than the evolution of evolvability. In parallel with the evolution of distinct strategies to cope with environmental change we identify evolved patterns of genome structuring. In genomes of lineages that evolved high evolvability the number of mutational targets for adaptation increases over time. In addition, a subset of these evolvable lineages evolves a distinct genome structure characterized by genes that remain unregulated, but whose dosage is rapidly tuned to the prevailing environment by duplications, deletions and point mutations.

## Results

**Fig. 1:**
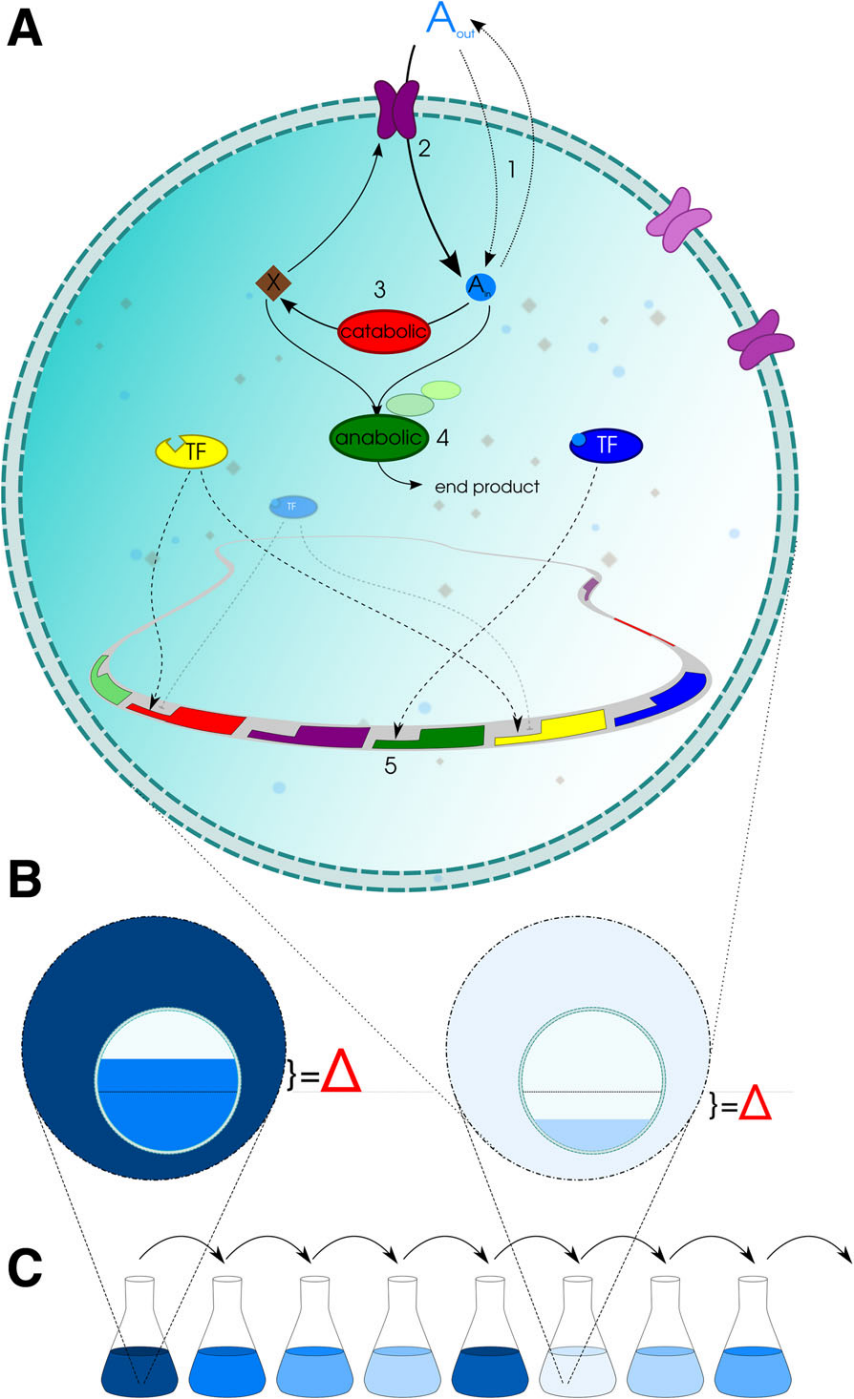
Virtual Cell model overview. **a** Virtual Cells have a circular genome that encodes metabolic and regulatory proteins. An externally available resource molecule (*A*) diffuses passively over the membrane (1) and is actively imported (2) by pump proteins. Once inside, *A* is converted to (*X*) by catabolic enzymes. *X* serves as the energy source for the import reaction (2). In addition, *A* and *X* are converted to an unspecified *end product* (4) by anabolic enzymes. Protein expression from genes (5) can be regulated by TFs if their binding motif matches the gene’s operator sequence. Binding of a ligand (*A* or *X*) by the TF alters its regulatory effect on gene expression. The genome can contain multiple copies of any of the gene types. Different copies may encode different values of the gene’s parameters, such as the enzymatic constants of the reaction that they catalyse or the binding motif and regulatory effect. **b** Fitness is determined by measuring the difference (*Δ*) between the realised steady state concentrations of internal *A* and *X* and the homeostasis target value (*dotted line*). **c** During the evolutionary experiments the external concentration of *A* is continually varying, while the homeostasis target remains constant. Cells have a chance proportional to their fitness to contribute offspring to the next generation

We employed the Virtual Cell (VC) model [21, 22] (Fig. 1) to study the evolution of adaptive strategies to cope with repeated environmental change. VCs exist in populations of fixed size and compete for a chance to produce offspring in the next generation, completely replacing the current population. Their fitness depends on the ability to maintain cellular homeostasis. Cells have a high fitness if they maintain equilibrium concentrations of the two internal molecule species *A* and *X* close to a fixed target during fluctuations in external resource (*A*) concentration that range over more than two orders of magnitude. Concentrations of *A* and *X* arise from the internal cellular dynamics that are given by a system of ODEs, representing the activities of the proteins in the cell. The activities of catabolic and anabolic enzymes and pumps directly affect concentrations of *A* and *X*. Transcription factors (TFs) influence gene expression when their binding motif matches a binding site in the operator of a gene. TFs can bind either *A* or *X* as a ligand, and have a differential regulatory effect on their downstream genes, depending on their ligand binding state. This ability to regulate gene expression depending on ligand binding state is crucial for the cells’ capacity to evolve homeostasis.

All proteins are transcribed from a spatially explicit, circular genome. Point mutations affect parameters of individual genes, such as the kinetic constants of enzymes, operator binding sites, and binding motifs and regulatory effect parameters of TFs. Large scale mutation events are the duplication, deletion or translocation of stretches of neighbouring genes as well as whole genome duplications (WGD). After duplicating, the two identical copies of a gene will diverge due to subsequent, independently accumulating point mutations. We are interested in the genome structure and mutational events on the line of descent (LOD) of a lineage (see “Constructing the line of descent” in “Methods”). In most of the analysis we focus on the mutational events fixed shortly before and after environmental change.

## Evolved wild-types rapidly adapt to novel environments

In a previous study we evolved 100 VC populations under fluctuating resource conditions [23]. From these we selected four WT-VCS that successfully evolved homeostasis regulation in their *native* environment for continued evolution in the current study. Here, we subjected populations to different periodic environmental changes at various change frequencies. The *novel* environments were constructed by changing *passive diffusion* rate of the *A* molecule, *degradation* rate of proteins and the stoichiometry of *conversion* from *A* to *X* of the catabolic reaction (Table 2 environments 1 and 2). Given that a change in any of these environmental parameters separately already has a significant effect on fitness, the combination of three simultaneously changed parameters represents a drastic change in environmental conditions, posing a significant challenge for cells to readapt. Note that the periodic environmental changes are discrete events and are in addition to the continuous fluctuations of the external resource concentration. Therefore, populations are constantly selected for homeostasis, while only periodically being exposed to a drastic change in the environmental conditions. Simulations were started from an early, intermediate and late evolutionary time point (see “Start populations” in “Methods”), with respect to the evolution of wild-types for all four WT-VCS. Each starting population is cloned to four fold replicates, that hence evolve under identical circumstances but with a different random number seed for mutations. Thus, our initial experimental set consists of 4 WT-VCS × 3 evolutionary time points × 2 environments × 4 replicates.

**Table 2:** Parameter values per environment

Environment	tar A	tar X	pas diff	con	degr	remarks
Native	1	1	0.1	4	1	Initial environment
Environment 1	1	1	0.4	8	0.5	Only *sensable* changes
Environment 2	1	1	0.05	2	4	Only *sensable* changes
Environment 3	1	1	0.1	8	1	Conversion only
Environment 4	1	1	0.1	2	1	Conversion only
Environment 5	4	4	0.05	4	1	WGD very rare
Environment 6	4	0.25	0.1	4	4	WGD never observed
Environment 7	4	1	0.4	4	4	WGD very common
Environment 8	0.25	0.1	0.1	8	4	WGD sometimes

Per environment the values of the homeostasis targets (tar A, tar X), *passive diffusion* rate, catabolic *conversion* stoichiometry and *degradation rate* are listed. The native environment was used for all populations in the initial phase of homeostasis evolution. The upper part of the table defines the *sensable* environments, in which *tar A* and *tar X* remain constant. In each evolutionary experiment populations are switched repeatedly between the native and novel environment. For an extended list of simulation parameters see Additional file 1: Table S1

**Fig. 2:**
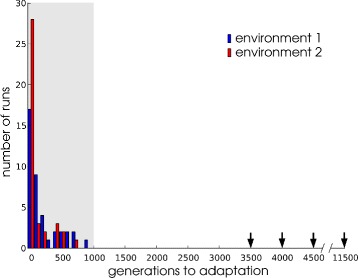
Time to readaptation. Pre-evolved WT-VCS were subjected to a change to a novel environment (1 or 2) and allowed 1000 generations (*grey shaded area*) to readapt. The time in generations to reach the high fitness value (>0.85) was recorded and binned per 50 generations. 4 WT-VCS × 3 evolutionary time points × 4 replicates yield 48 runs for each environment. 83% and 86% of populations in environment 1 and 2, respectively, reached high fitness before 1000 generations. For comparison, the adaptation times of the original WT-VCS are indicated by arrows in the same plot

Figure 2 shows readaptation times after the first round of environmental change, defined as the time required to reach a fitness >0.85, to the novel environment. As a comparison, the number of generations that the four WT-VCS required to initially evolve homeostasis are also shown. Strikingly, readaptation to the novel environment was much faster than the initial adaptation. This was despite the initial drop in fitness to ≈0. (data not shown) when the novel environment was first presented. This result is in agreement with our findings in a previous study of a larger set of populations and environments [23]. It suggests that the evolved capacity for homeostasis regulation endows lineages with higher adaptability in novel environments.

## Evolution of evolvability at low frequency environmental change

Next, we continued the evolution of the populations by periodically changing between their native environment and a novel environmental condition. Previous research has shown that both genome organization [18] and network structure [19, 24] can evolve to accommodate rapid phenotypic switching between two different environmental targets. Here, both levels of organization can potentially evolve. In contrast to these previous studies, here, VCs can also *sense* the effects of environmental change, analogous to the sensing of resource fluctuations used for homeostasis regulations. This gives us the opportunity to study the interplay of the evolution of regulation and evolvability. To do so, we performed evolutionary experiments where WT-VCS adapted to periodically changing environments.

In our first setup we let environments change every 1000 generations. We find that over the course of 14000 generations of evolution the majority of replicates (environment 1: 52% ; environment 2: 75%) at some point fail to reach a fitness >0.85 within the 1000 generation environmental epoch. In other words, these lineages lose the ability to maintain resource homeostasis after environmental change. Strikingly, if a population fails to readapt to a change, the success rate on the subsequent change goes down to 55% compared to a 93% success rate if the population previously readapted successfully. The failure to readapt may be a consequence of losing the gene regulatory network (GRN) structures that are essential for homeostasis regulation with respect to nutrient fluctuations. As is evident from the much longer time scales for initial evolution (Fig. 2), homeostasis regulation is comparatively hard to evolve de novo. Therefore, one explanation for the repeated failure to readapt to environmental changes of some lineages would be that these at some point lost the regulatory structures in their GRN that functioned to maintain resource homeostasis. It would then take a considerably longer evolutionary time to evolve the lost genetic information anew. This is comparable to the initial phase of evolution, where homeostasis took a long time to evolve (see arrows in Fig. 2). In contrast, lineages that retain the regulatory structures needed for resource homeostasis would have a shorter evolutionary path to becoming adapted to the other environment.

**Fig. 3:**
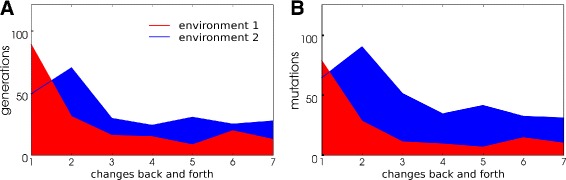
Generations and mutations needed for readaptation in successfully adapting lineages. Populations under environmental change at 1000 generation intervals between the native and novel environment. As novel conditions, environments 1 and 2 defined in Table 2 were used. The number of generations (**a**) and mutations (**b**) needed to readapt is plotted at every back and forth change of the environment. The values are averaged over all WT-VCS replicate runs in the respective environment that are not ‘evolutionary dead-ends’. (see Additional file 1: Figure S1 for a larger set of environments)

**Fig. 4:**
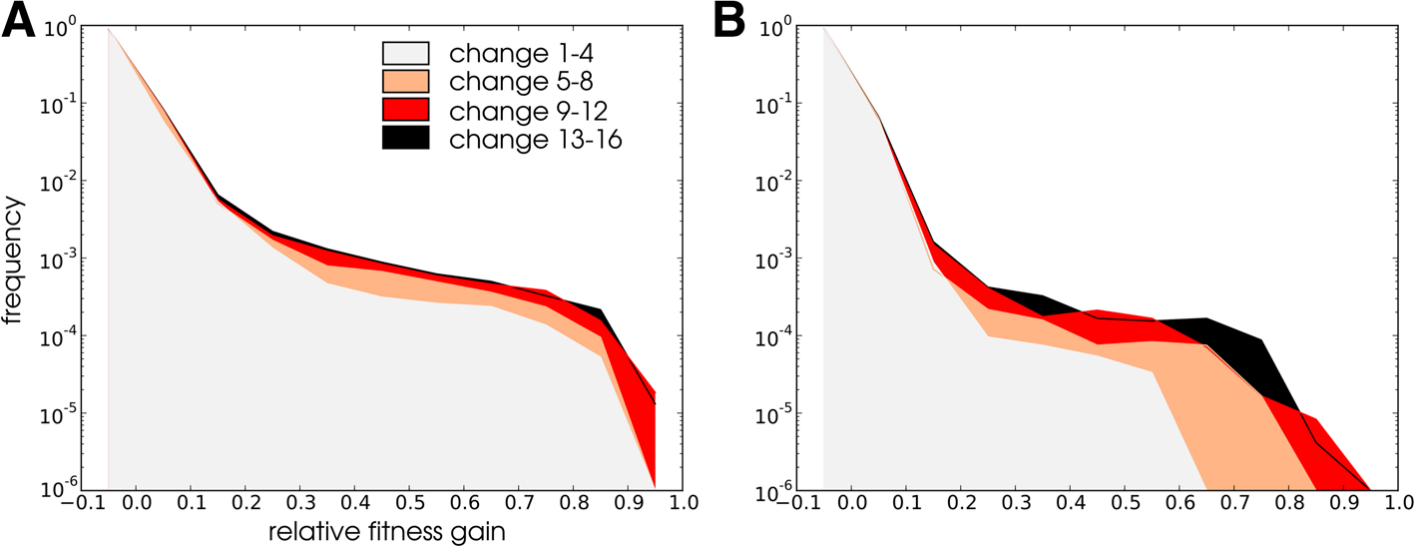
Evolution of mutational landscape towards beneficial mutations. We put individuals in the LOD that lived just before a change of environment (see Methods ancestor tracing) in the next environment and let them undergo a single round of mutation. In this alternative environment we measure their fitness before and after the random mutation and define the fitness effect of the mutation as the gain relative to the maximum possible gain (absolute gain/(1−fitness before mutation)). Different colour graphs are for different periods of the evolutionary simulations. For environment 1 (**a**) and environment 2 (**b**) measures for all populations and all replicate runs are averaged

The lineages that fail to readapt could be considered as ‘evolutionary dead-ends’. When we removed them from the analysis and focus on the successfully readapting lineages we find that both the time in generations (Fig. 3 a) and the number of mutations (Fig. 3 b) needed to readapt decrease. Clearly, the populations become more evolvable with respect to the periodic environmental change. To better understand how populations were able to adapt more rapidly to environmental change over time we analysed the potential for mutations to reinstate fitness following environmental change. This analysis was applied along the LOD as follows: Each LOD was divided into four equal length time frames and in each time frame individuals from LODs in all simulation were combined. For each individual we then recorded the fitness measured in the (unseen) alternative environment before and after a single round of mutation, thus assessing the adaptive potential of mutations in the environment that individuals did not experience during their life time. We find that the fraction of large benefit mutations increases over successive evolutionary time frames (Fig. 4 a, b), explaining why these populations need fewer generations and mutations to adapt later in the experiment. Although the fraction of these mutations is low within an individual (in the order of 10^−5^−10^−3^) there is a large chance that a mutation recovering most of the fitness will arise in the populations within a few generations. Thus, the increased evolvability towards periodic change is due to changes in the mutational landscape that becomes biased towards mutants that are fit in the alternative environment.

**Fig. 5:**
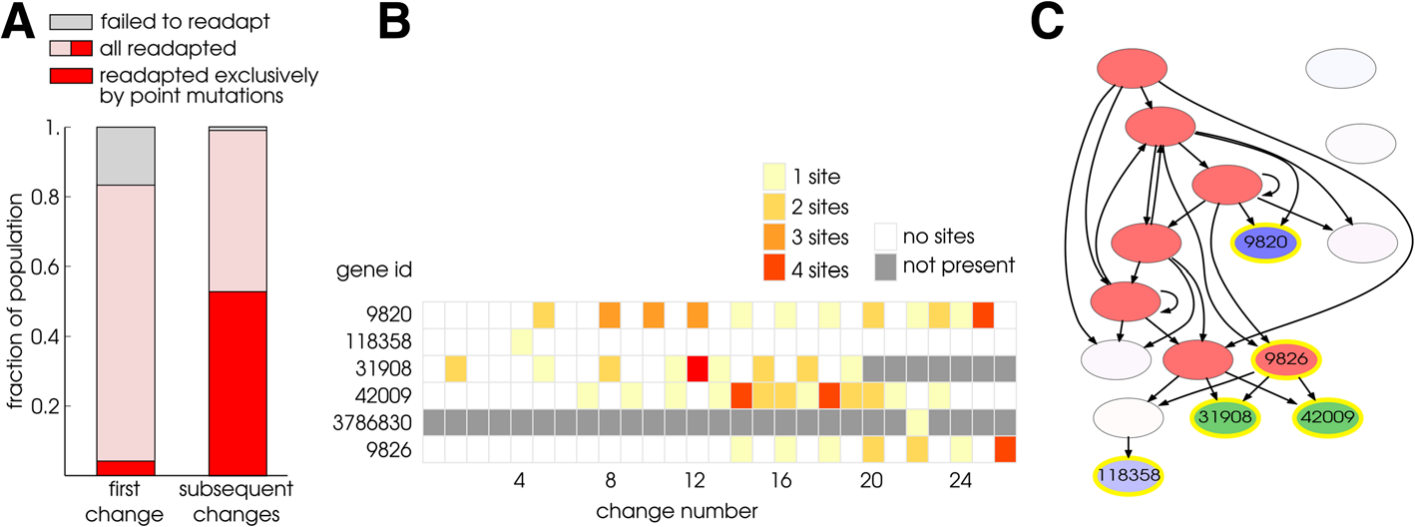
Mutational priming in the GRN. **a** Fraction of changes where populations in environment 1 needed only point mutations to readapt. This was measured by examining mutations found in the LOD, from the generation immediately after a change occurred until an individual with fitness >0.7 was found. The first pair of changes back and forth is compared to all subsequent changes. **b** At the time points before each (numbered) environmental change we plot, for individuals in the LOD of a sample evolutionary run, the number of primed sites of all the genes in the genome. We systematically mutate all parameters (sites) of all genes in the genome. If mutating a site can restore fitness to >0.7 in the alternative environment then this site is a primed site of a gene. A gene can have more than one site (parameter) that is primed, as indicated by the colour of the squares. Only genes are plotted that have at least one primed site at some point during the evolutionary simulation. Genes that come into existence only later, after duplication, or disappear due to deletion, are partly greyed in the plot. **c** shows the GRN at the halfway point of the simulation (change 13) where genes with ‘primed sites’ are indicated by *yellow outlines*. Note that at a subsequent evolutionary time point enzyme 31908 is deleted from the genome and enzyme 3786830 exists only transiently in the genome between change 22 and 23. *Red*: TFs, *green*: catabolic enzymes, *blue*: anabolic enzymes, *purple*: pumps. *Arrows* indicate regulation (both positive and negative) by TFs. The colour saturation indicates gene essentiality, measured as the relative loss in fitness loss upon knockout (deletion) of the gene. Note that the colourless gene is in fact a TF with very low essentiality value.

Another way to infer the effect of genome restructuring on the evolution of evolvability is by looking at the type of mutations that become fixed during readaptation. For lineages that undergo changes to novel environment 1 we recorded the set of mutations occurring immediately following an environmental change until they recovered fitness to >0.7. Comparing the first pair of changes to the novel environment and back to the native environment with all subsequent changes we find an increase in the importance of point mutations for fitness recovery (Fig. 5 a). Whereas initially only 10% of readaptations exclusively use point mutations, this increases to 50% during subsequent changes. Figure 5 b shows for one simulation the evolution of ‘primed sites’ of genes that allow fitness to be recovered with a single mutation. Heat map colours indicate for how many distinct parameters (sites) a single mutation to a new value can lead to instant recovery of fitness. In the example, the number of genes as well as the number of target sites per gene is going up during the first part of the simulation. In the GRN in Fig. 5 c genes are highlighted that contained such primed sites at some point in the evolutionary. It can be seen that enzymes as well as TFs can evolve primed mutational sites. Thus, the mutational landscape evolves to connect genotypes encoding the different phenotypes most suited for the different environments.

We conclude, firstly, that populations that maintain general regulation of homeostasis are surprisingly evolvable when challenged with novel environmental conditions. Secondly, that these populations further increase the speed of adaptation by evolving primed mutational sites, that allows rapid phenotypic switching. And thirdly, that this process is remarkably efficient, given that environments change only once per 1000 generations.

## Time scales of evolvability and regulation

General homeostasis regulation in WT-VCS appears to confer high evolvability when populations encounter novel environmental conditions. Moreover, evolvability increased further when environments changed only once per 1000 generation. When changes to the environment are more frequent, it is expected that phenotypic plasticity becomes increasingly advantageous [25] as it avoids the waiting time for beneficial mutations. To study the transition from evolutionary to regulatory adaptation we expanded our analysis to environmental changes at every 100, 50, 30, 20 and 10 generations. In addition, to increase the scope for the evolution of regulated phenotypic plasticity we study evolution in two simplified environments (Table 2 environments 3 and 4), where catabolic conversion stoichiometry is the only parameter that changes (increased in environment 3 and decreased environment 4). For this analysis we restrict the starting population set to two randomly selected WT-VCS, that were propagated from the intermediate evolutionary time point. Now that changes are in quicker succession, we retain lineages that fail to adapt to some changes in this part of the analysis.

**Fig. 6:**
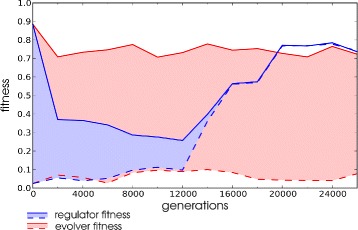
Regulator and evolver strategies. Fitness values over evolutionary time measured in the environment that the LOD historically experienced (*solid lines*) and the alternative environment (*dashed lines*). Data points are averages over the first 30 generations after switching and bins per 2000 generation intervals

Under higher frequencies of environmental change two adaptive strategies emerge. Figure 6 illustrates the distinct fitness profiles of these strategies in two simulations. For the LOD fitness is determined both in the *actual* environment that an individual experienced and the *alternative* environment that it did not see during its life time. The first strategy, which we named an *evolver* strategy, shows dramatically different fitness values between the experienced and the alternative environment. It follows that every time the environment changes, the lineage has to readapt by evolutionary adaptation. Moreover, the evolver strategy emerges nearly instantaneously and is maintained for many changes throughout the simulation. In contrast, a *regulator* strategy evolves much more slowly, with low levels of fitness in both the actual and the alternative environment and is further characterised by slowly converging levels of fitness in the actual and alternative environment. This convergence is due to phenotypic plasticity of individuals, that adaptively regulate gene expression to match each environment. Having observed these clearly distinct adaptive strategies we came up with criteria to classify different simulations as using either of these strategies. Lineages were classified evolvers from the moment that they successfully readapted from a drop in fitness <0.4 to a fitness >0.7 within 10 generations of environmental change, in at least 9 out of each 10 consecutive changes. They were classified as regulators from the moment that they attained a fitness >0.7 in both environments at every subsequent generation.

**Fig. 7:**
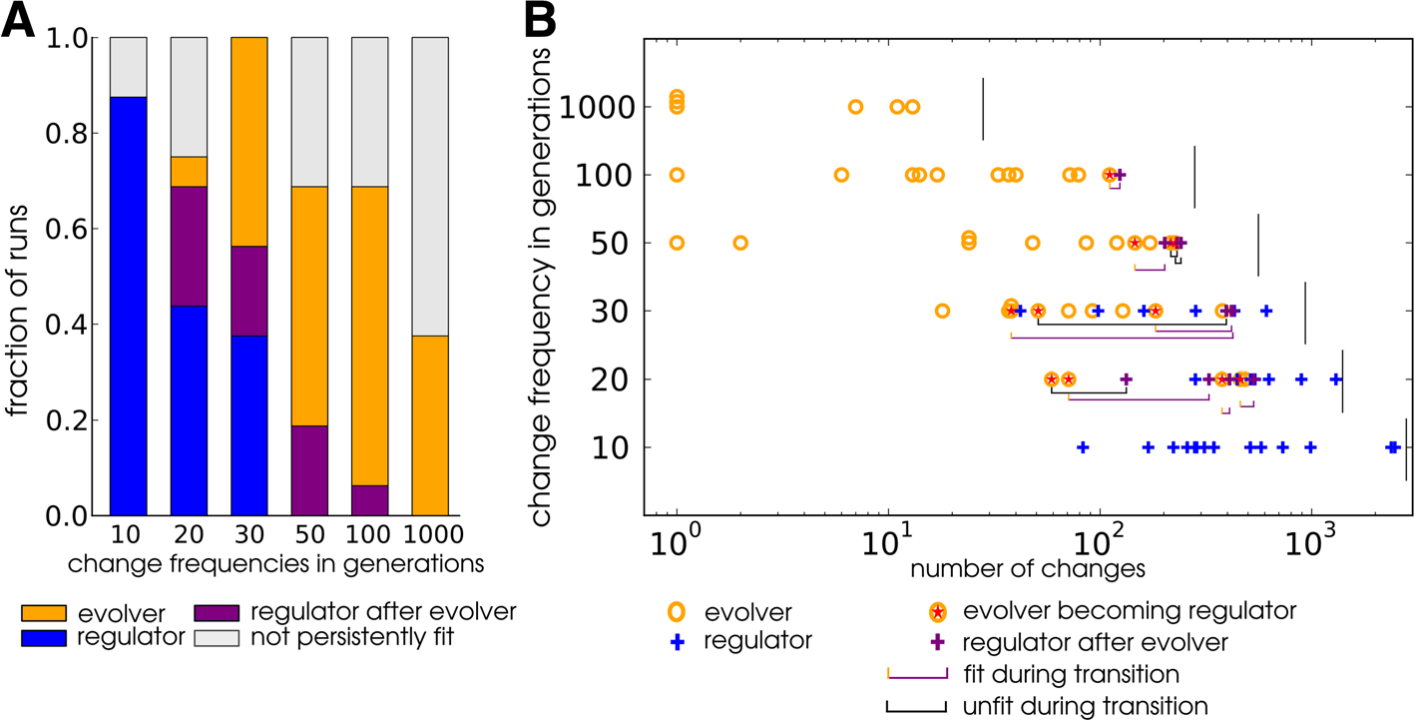
Evolution of adaptive strategies at different ecological time scales. Data from simulations in environments (1,2,3 and 4) are combined, thus totalling 2 WT-VCS × 4 environments × 2 replicates = 16 runs per change frequency. The fractions (**a**) and time of appearance (**b**) of the evolver and regulator strategies in the LOD are plotted for evolutionary simulations under different frequencies of environmental change. The first occurrence of a regulator strategy is recorded if the individuals in the lineage maintain a fitness value >0.7 in both the native and novel environment. The first occurrence of the evolver strategy is recorded if for 9 out of 10 subsequent changes the fitness first drops below 0.4 and then rises above 0.7 within 10 generations of environmental change. If an evolver strategy arises that subsequently gives rise to the regulator strategy in the same lineage, this event is additionally marked in B with a *red star* and a line indicating how long and how (*purple*: no fitness loss, *black*: temporary failure to reach fitness criterion) the transition to the regulator strategy proceeds

**Fig. 8:**
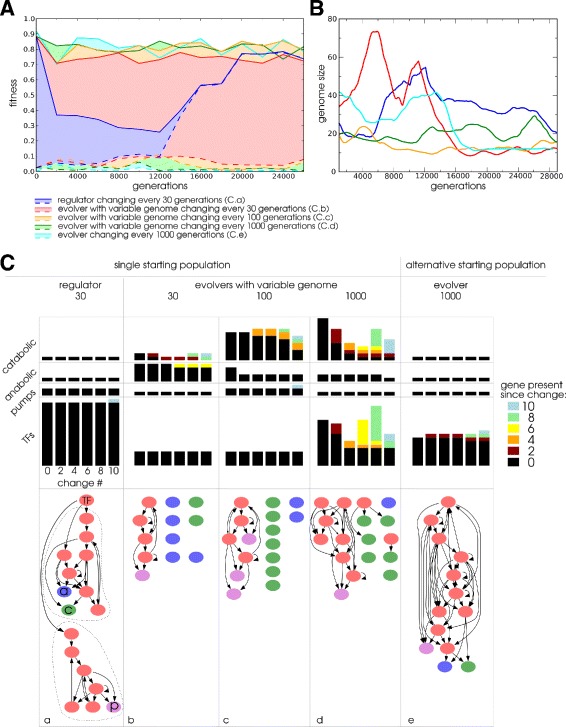
Evolutionary fitness and genome dynamics of example lineages in environment 3. All data shown are from a set of five lineages that evolved under conditions changing between the native and novel environment 3. Lineages were selected to illustrate distinct evolver and regulator fitness and genome dynamics. Two of the lineages evolved under 30 generation periodic changes, one at 100 generation period and two more under a 1000 generation period. Four out of the five lineages evolved in different simulations from a single WT-VCS. One of the lineages that evolved under 30 generation periodic change has the evolver strategy (*red lines* in **a** and **b**), while the other lineage under the same conditions evolved a regulator strategy (*blue lines*). **a** Fitness values over evolutionary time measured in the environment that the LOD historically experienced (*solid lines*) and the alternative environment (dashed lines). Data points are averages over the first 30 generations after switching and bins per 2000 generation intervals. **b** Genome size in the LOD. Line colours correspond with colour coding in **a**. In each LOD this segment corresponds with 10 environmental changes. **c** The top graphs show the conservation of genes in the final ten environmental changes. Bars represent number of genes per gene category and are coloured coded according to time of origin (counted in environmental changes) of genes. Only changes in the last ten changes in the simulation are shown, corresponding to 300, 1000 and 10000 generations, respectively for period 30, 100 and 1000 environmental changes. Bottom graphs show the essential gene regulatory network at the final time point of the simulation. *Red*: transcription factors, *blue*: anabolic genes, *green*: catabolic genes and *purple*: pumps. (C.a) Two regulatory modules that do not feed back on each other have been highlighted. This GRN structure fulfils the modularity criterion (see main text)

Using this classification, Fig. 7 a shows the fraction of simulations that evolved one of the strategies for different frequencies of environmental change. When environments change once per 1000 generations, only the evolver strategy evolves. As expected, regulators become more frequent when environments change more frequently. At intermediate frequencies both strategies evolve and persist. At the highest change frequencies none of the lineages are classified as evolvers. Notably, some lineages that are evolvers early in evolution, later transition to the regulator strategy. The evolution of strategies in the opposite direction was, however, never observed. Interestingly, evolvers at lower frequencies always have a higher fitness, measured in the first 30 generations after a change (because this is the highest change frequency where evolvers are found), than those evolved at higher frequencies (Additional file 1: Table S2 and Fig. 8 a). Possibly, spending a longer period of time in a single environment allows better fine tuning of homeostasis regulation (note that resource concentrations fluctuate continuously). Alternatively, the frequent population bottlenecks when very few fit mutants contribute offspring to the next generation during the frequent environmental changes could put a strain on the maintenance of genetic information.

We then analysed the relationship between change frequency and the time scales at which either strategy evolved. In Fig. 7 b we plotted the time of establishment of either strategy. The evolver strategy can evolve remarkably fast, sometimes establishing immediately at the first environmental change. In a strongly contrasting pattern, regulation invariably takes many changes and generations (Fig. 7 b and Additional file 1: Figure S2) to evolve. Also, whenever both strategies co-occur at a given frequency, the evolver strategy is established prior to any of the regulators. We conclude that evolvability can be a highly efficient strategy for populations to continually adapt to changing environmental conditions. Only when the periodic environmental change occurs with a sufficiently high frequency and over many iterations can populations reach a comparable level of adaptation with regulated phenotypic plasticity.

### Contingency and robustness of strategies

**Fig. 9:**
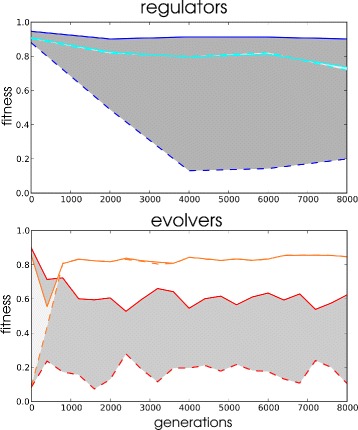
Robustness and plasticity of evolvers and regulators. Example evolutionary simulations of two regulators (*top*; *blue* and cyan) that had previously evolved under 10 generation periodic changes and were subjected here to 1000 generation periodic changes and two evolvers (*bottom*; *red* and *orange*) that had previously evolved under 1000 generation period changes and were subjected here subjected to 10 generation periodic changes. Continuous and *dashed lines* are fitness in the actual and alternative environment, respectively, with the difference filled in (*grey*)

Sometimes biological replicates of a single WT-VCS start population subjected to identical change frequency evolved different strategies (e.g. Fig. 8 a blue and red trajectories). These lineages maintained their respective strategy for over 10000 generations, showing that the strategies can have comparable success rates at evolutionary time scales and are contingent on their prior evolutionary history. Nonetheless, we have seen that the evolution of the two types of adaptive strategy clearly depends on the frequency of environmental change. We wondered how robust each strategy was, once it had evolved, under conditions that would otherwise favour the evolution of the alternative strategy. We selected evolvers that evolved under the 1000 generation change regime and regulators evolved under the 10 generation regime and subjected each to the opposite change frequency, namely 10 generation changes for the evolvers and 1000 generation changes for the regulators. Because we suddenly apply an environmental change frequency at the opposite side of our frequencies range, this can be considered a ’severe case’ for testing robustness. Interestingly, we found that the evolutionary response to a change in frequency can be twofold. Figure 9 illustrates that both evolver and regulator populations may react by either evolving the alternative strategy or maintaining their prior strategy. We conclude that both strategies can enable a population to adapt persistently under a wide range of frequencies of environmental change. Nevertheless, regulation provides superior fitness when the environment changes rapidly. Importantly, even though smooth mutational paths exist between the two strategies for a subset of the evolved solutions, in other cases lineages appear stuck in the strategy that they evolved earlier.

## Genotypic strategies

So far, we identified different strategies by analysing the phenotypes of ancestors in different environments. Now, to get more mechanistic insight into regulators and evolvers we study the structure and evolution of their genome and gene regulatory networks. Large fluctuations in genome size occur over the course of evolution (Fig. 8 b). Fluctuations are most pronounced during the first half of the evolutionary experiment, while streamlining dominates the second half [22]. These dynamics are expected to initially increase mutation rates and variation and subsequently reduce mutational load, when the role of adaptive evolution diminishes [22, 26, 27].

Because the large genomes and tangled networks during early evolution pose a problem for in analysing the function of the GRN, we apply a pruning step to remove redundant genes. (see “Functional genome analysis” in “Methods”). In the simplified networks we find recurrent evolution of a characteristic GRN structure among evolvers (22%). The structure is characterised by a regulated part that controls expression of one or two of the protein types (catabolic and anabolic enzymes and pumps) in the metabolism and an unregulated part in which proteins of the remaining mostly exist in multiple copies. The unregulated, variable part of the genome undergoes regular duplication and deletion to adjust protein dosage on demand, while the core regulated part of the genome is largely conserved (Fig. 8 and Additional file 1: Figure S3).

Some of the GRNs of regulators evolved a remarkable, modular structure, characterized by independent subsets of TFs exclusively regulating a specific enzyme type. To investigate this observation further we tested for the presence of multiple interconnected gene sets (modules), that do not have regulatory feedback to the other modules. Moreover, these modules should segregate the regulation of one of the enzyme types from the others. Applying these criteria we found that in the full change environments (1 and 2) 27% of regulators evolve a modular network structure. Intriguingly, in the environments where only the catabolic conversion stoichiometry changes (3 and 4), modularity evolved in 94% of lineages (e.g. Fig. 8 a). This suggests that modularity is a highly evolvable feature whose evolution depends on the specific type of environmental change. Because the stoichiometry of the reaction from *A* to *X* changes in the simplified environments environments it can be beneficial to independently regulate catabolic and anabolic enzymes that control the internal balance between *A* and *X*. In conclusion, we find that evolver and regulator strategies are associated with particular GRN structures and evolutionary genome dynamics in a significant subset of lineages.

## Discussion

Adaptation to changes in the environments is vital for long term survival of populations. Unsurprisingly, species have evolved many molecular mechanisms to regulate their phenotype in order to adapt to environmental changes [4, 28]. However, it is increasingly evident that evolutionary adaptation can be a fast and efficient mechanism for populations to adapt to novel circumstances [8, 16, 29, 30]. Here, we have shown that rapid evolutionary adaptation to novel environmental changes is expected when organisms have previously evolved gene regulatory structures for maintaining homeostasis under external nutrient fluctuations. Moreover, when the environment changes periodically, populations evolve an even higher degree of evolvability with respect to the change, requiring few mutations to readapt. Remarkably, this effect is already present after a few changes that are 1000 generations apart.

When environmental change becomes more frequent, the selective advantage of a fully regulatory response to change increases. In line with expectation we found that an increasing fraction of populations evolved regulated phenotypic plasticity towards both environments, when change frequency increased. Interestingly, a recent study found that yeast evolved better phenotypic plasticity towards two stressful environments during 300 generations of evolution in rapidly alternating (10 generations) conditions [12]. Here, by investigating adaptation on a continuum of ecological time scales we could capture the transition between highly effective mutational adaptation and hard to evolve but very stable physiological regulation. Remarkably, during the evolution of the latter strategy, lineages would frequently evolve evolvability first. A related phenomenon has been observed in yeast species that initially adapted by transiently duplicating chromosomes or chromosomal segments [16, 31], before evolving fine tuned regulation in a novel environmental condition [16] (but see [32]). While we found that some lineages displayed evolutionary plasticity by transitioning between strategies, other populations robustly maintained their previously evolved strategy in a new environmental change regime that was more favourable to the alternative strategy without losing viability (Fig. 9).

### Evolution of genome structure

In populations that used the evolver strategy we observed a recurring network structure characterised by a highly connected and stable part regulating a subset of the enzyme types (core), complemented with an unregulated parting containing the remaining enzyme type(s) at variable copy numbers (variable genome). This structuring may either be selected to enhance adaptability or, alternatively, could neutrally emerge due to long term evolution in periodically changing environments. For example, having a set of unregulated copies of a particular enzyme enables rapid adaptive dosage adjustments by gene duplication and deletion. Conversely, this structure may be the result of a ratchet effect due to repeated bottlenecks [33], that gradually breaks up the existing regulatory structures. The operation of a ratchet is supported by the observation that evolvers in rapidly changing environments, that experience more and faster bottlenecks have lower fitness than when change is slow. The resulting structure is more likely to provide a fast and relatively robust mode of readaptation. Therefore, we hypothesize that the evolution of a *core-variable genome* structure is due to the interplay between both mechanisms. Finally, a generic genome streamlining pattern [6, 22, 34] that we also observe in our current experiments (Fig. 7 b), combined with the evolution of a sparsely regulated core-variable genome may cause a population to become evolutionarily stuck, as seen for evolvers that maintained their strategy under a change regime that otherwise strongly selected for the regulator strategy (Fig. 9).

In contrast to core-variable genome evolution, modularization of GRNs in regulators appeared significantly environment specific, evidenced by the much higher fraction of modular networks encountered in populations changing to the simplified environments 3 and 4 compared to the full environments 1 and 2. Although modular network structures have previously been shown to evolve in environments consisting of modular sub-goals [35, 36], here the underlying process appears qualitatively different, because environments were not constructed as modular sub-goals or presented in a modular way. That is, we modelled environmental changes as a discrete, integral change in environmental parameters. In our case, GRNs therefore appear to evolve a representation of the modularity inherent in the metabolic task, by segregating the functional sub-networks used for specific metabolic reactions, thereby capturing the structure of their outside environment without for presenting these reactions in a modular fashion [37–39].

### Divergence and evolutionary rates

The diversity of evolved strategies in our simulations could exist under identical environmental conditions, showing that they confer comparable fitness. Remarkably, evolver and regulator strategies sometimes evolved from an identical genetic background (Fig. 8). Once evolved, both the modular genome structures of regulators and core-variable genome structures of evolvers were stably inherited over long evolutionary time frames. Yet, the rate of genomic change for the evolver strategy is expected to be much higher than that of the regulator. Without any additional knowledge, these differences in evolutionary rates could well be interpreted as the result of differences in external selection pressures on the two closely related populations. This example shows that any hypothetical inferences about environmental conditions experienced by a population are contingent on the adaptive strategy that it employs. Put differently, identical environmental conditions can be perceived as stable by one populations, while demanding constant evolutionary adaptation in another.

### Loss of homeostasis regulation

A significant subset of simulations failed to regain fitness at some point after environmental change. If these populations are allowed to continue evolving, their subsequent adaptability is significantly worse. Thus, losing pre-evolved homeostasis regulation decreases the chance to adapt to subsequent environmental changes. Having homeostasis regulation for nutrient fluctuations as the general fitness criterion for VCs played an important role in our result of finding high adaptability and evolvability. Arguably, homeostasis is a vital survival mechanisms in natural populations. In fact, much of the fast readaptation to novel conditions observed in laboratory conditions exploits pre-existing adaptation mechanisms [8, 16, 39], by mutationally inducing gene over or under-expression. This feature makes our model qualitatively different from most other modelling approaches, that do not consider such a pre-evolved survival mechanism in all environments [18, 19, 35, 40], and uniquely enabled us to observe the interplay between evolvability and regulation.

### Sensing changes

**Fig. 10:**
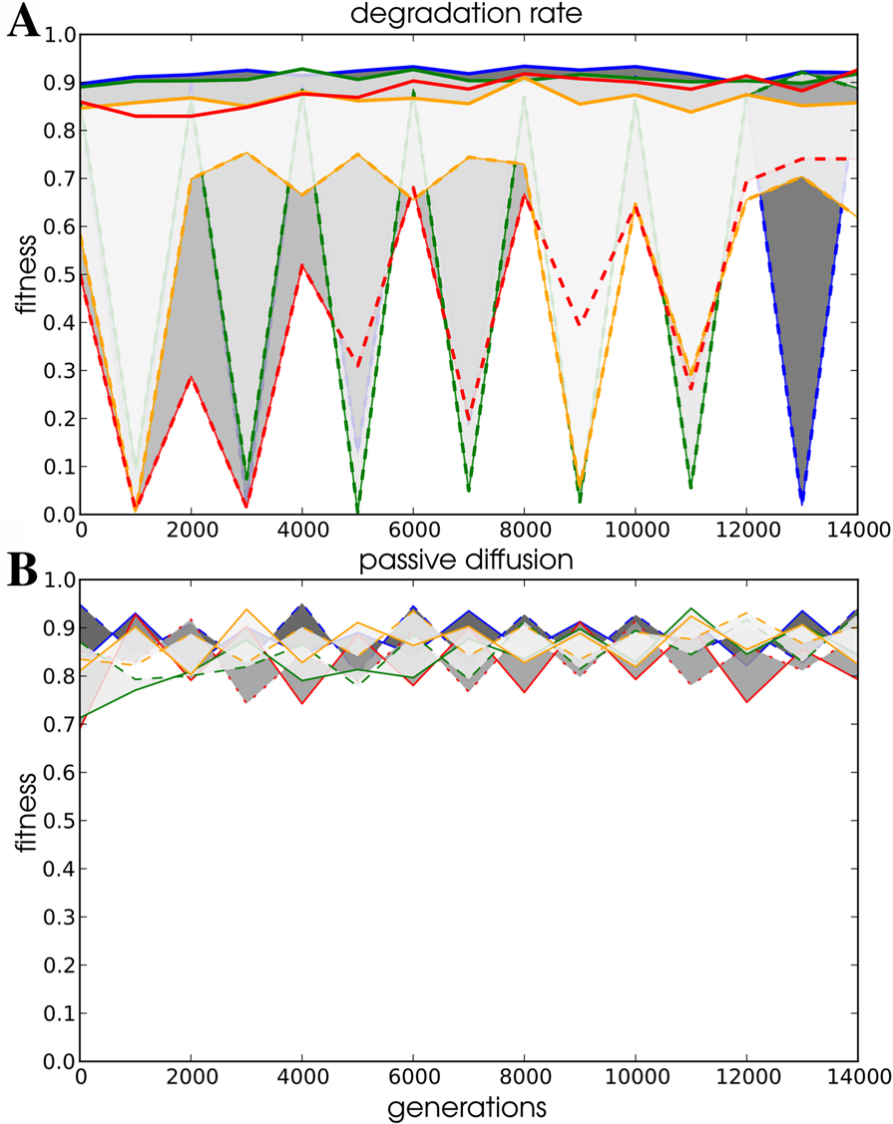
Single parameter environmental change. Fitness in the current (*solid line*) and alternative environment (*dashed line*) measured in the LOD. Each fitness measure is the average of the first 30 generations after an environmental change. **a** Four populations adapting to changes in passive diffusion. Passive diffusion is increased like in environment 5. **b** Four populations adapting to changes in degradation. Degradation is decreased like in environment 5

During the evolution of homeostasis regulation under external resource concentration changes spanning two orders of magnitude, pumps and enzymes become tightly regulated by TFs that can sense internal concentrations of the nutrient (*A*) and energy carrier (*X*) molecules. The regulatory control mechanisms that evolve for homeostasis maintenance can be exploited during adaptation to certain dimensions of environmental change. For example, VCs show a high degree of preadaptation to changes in passive diffusion and more varying levels of preadaptation to changes in degradation rate (Fig. 10). Similar results were found during experimental evolution of bacteria [13, 41, 42] and yeast [43] to various environmental stressors, where evolved lines were often fitter or produced more biomass than ancestor and control lines under previously unseen stress conditions.

Living organisms constantly monitor their internal metabolite concentrations via ligand binding and other sensing mechanisms. In this study we focused on environmental changes that cells can perceive directly or indirectly by internal sensing, mirroring the majority of adaptive evolution in the wild. In contrast, most previous computational work on adaptation to environmental changes has either imposed novel fitness targets, without a mechanism to sense the change [19, 40, 44] or employed short-cuts to sensing via an input node in a regulatory network that represents the environmental condition directly [38, 45]. Although we cannot easily mimic the latter type of sensing, it was possible to impose changes of the fitness criterion that VCs cannot sense. Additional experiments with such *non-sensable* changes in the homeostasis fitness criterion (Table 2 environment 5 and 6) indicated that adaptation takes much longer, but that evolvability still noticeably improved in most cases (see Additional file 1: Figure S4). One reason for the longer adaptation times, may be that, by definition, populations cannot evolve phenotypic plasticity to non-sensable changes, because they lack a signal to initiate regulated physiological change. Nevertheless, the genotype to phenotype map can still evolve to allow for efficient phenotype switching with little genotypic change [19].

In contrast, here, we observe adaptation to changes on a continuum of ecological time scales. The resource fluctuations that VCs continuously experience during their life time represent the fastest frequency on this scale. The homeostasis regulation that VCs evolve to cope with these fluctuations put them at an advantage when they are challenged with novel other types of environmental change at slower time scales. Similarly, for natural populations we expect that general homeostasis mechanisms can be exploited for subsequent adaptation to more drastic environmental perturbations of cellular dynamics.

Lastly, it is important to realise that natural populations use several adaptive mechanisms and strategies that were not modelled in VCs. Bacterial populations undergo frequent horizontal gene transfer, acquiring environment specific genes that exist in the pan-genome when needed [46]. In addition, mutator strains with up to three orders of magnitude higher mutation rates arise frequently under conditions where adaptive exploration of the mutation landscape is beneficial [47]. Perhaps most intriguingly, the budding yeast has recently been found to posses a regulatory pathway that induces directed mutations under specific environmental circumstances [48]. The last example clearly indicates that regulation and mutation are far from strictly separate adaptive strategies. Rather, the need for robustness and evolvability synergistically shape the genotype and phenotype of a lineage, as information about the changing environment is integrated over long evolutionary time scales [49]. Notwithstanding the additional strategies that species may employ to cope with a changing environment, we believe the current study provides useful insights into antagonistic and synergistic evolutionary patterns when allowing phenotypic plasticity and evolvability to evolve simultaneously. Moreover, the, to our knowledge, unique requirement in our model to maintain homeostasis, an ubiquitous regulatory mechanism in biology, while adapting to environmental change, adds to the relevance of our findings for understanding adaptation in real populations.

## Conclusions

When challenged with novel environmental conditions, populations can show remarkably fast recovery, by exploiting previously evolved homeostasis regulation. In addition, when the environment changes periodically populations evolve a highly efficient evolver strategy, by increasing the number of genotypic targets for beneficial mutations. As the frequency of changes increases, the evolver strategy gives way to evolution of regulators, albeit at much longer evolutionary time scales. Remarkably, both types show evolutionary plasticity with respect to transitioning to the other strategy. At the same time we found cases of evolver as well as regulator lineages that robustly retained their respective strategy under change frequencies that would otherwise promote evolution of the alternative strategy. Moreover, both strategies could evolve under identical conditions and genetic background, demonstrating high contingency of the evolutionary trajectories. We conclude that organisms can exploit regulatory mechanisms evolved for fast homeostasis regulation when adapting to periodic environmental changes. As a result, adaptation by mutations can compete successfully with regulation over a continuum of ecological time scales. Regulation only evolves when environmental changes are very frequent and continue over long evolutionary time scales.

## Methods

The Virtual Cell (VC) model is an individual based model implemented in C++ and available online at https://bitbucket.org/thocu/virtualcell. The population size is constant (1024 individuals by default) and at each generation all individuals are replaced by new offspring. The reproduction chance is fitness proportional, based on the ability to maintain homeostasis while the external resource concentration is fluctuating.

### Internal dynamics

The internal dynamics of VCs are governed by two metabolite species and four types of proteins. The first metabolite is a resource molecule (*A*) that is present both externally and internally. The second is an energy carrier molecule (*X*) that is only present internally, needed for active transportation of *A* by pump proteins.

**Diffusion** *A* diffuses over the membrane passively with a rate, *Perm*:

1 $$ \frac{d[A]}{dt} = \left(\left[A_{out}\right]-[A]\right)\cdot Perm   $$

**Protein expression and degradation** Proteins are transcribed at a basal rate determined by their gene’s promoter strength *Pr*. Basal expression can be affected positively and negatively by regulation factor *Reg*. All proteins are degraded at a fixed rate *Degr*:

2 $$ \frac{d[{Prot}]}{dt} ={Pr} \cdot {Reg} - {Degr} \cdot [{Prot}]   $$

In addition to the promoter site, a gene encodes a binding site, and parameters for kinetic constants in the case of enzymes and pumps or a binding motif in the case of transcription factors (TF).

**Pumping** Pumps, consume energy carrier molecules to pump external resource molecules into the cell at a maximum rate $\phantom {\dot {i}\!}V_{{max}_{p}}$. *K*_*A-p*_ *Ka*_*p*_ and *Kx*_*p*_ are the binding constants for external resource *A*_*out*_ and energy molecule *X*, respectively:

3 $$ \frac{d[X]}{dt} =\frac{-d[A]}{dt}   $$

4 $$ \frac{d[A]}{dt} =\frac{\left[A_{out}\right] \cdot [X] \cdot V_{{max}_{p}} \cdot \left[{Prot}_{p}\right]}{\left(\left[A_{out}\right] + {Ka}_{p}\right)\left([X] + {Kx}_{p}\right)}   $$

**Catabolism** Catabolic enzymes, *prot*_*c*_, convert internal resource concentration into energy *N* carrier molecules at a maximum rate $\phantom {\dot {i}\!}V_{{max}_{c}}$. *Ka*_*c*_ is the binding constant for internal *A*:

5 $$ \frac{d[A]}{dt} =\frac{-\left[{Prot}_{c}\right] \cdot [A] \cdot V_{{max}_{c}}}{[A] + {Ka}_{c}}   $$

6 $$ \frac{d[X]}{dt} =-N \frac{d[A]}{dt}   $$

**Anabolism** Anabolic enzymes, *Prot*_*a*_, convert internal *A* and *X* into a non-reactive product at a maximum rate *Vmax*_*a*_. This product does not affect fitness.

7 $$ \frac{d[A]}{dt} =\frac{-\left[{Prot}_{a}\right] \cdot [A] \cdot [X] \cdot {Vmax}_{a}}{\left([A] + {Ka}_{a}\right)\left([X] + {Kx}_{a}\right)}   $$

8 $$ \frac{d[X]}{dt} =\frac{d[A]}{dt}   $$

**TF ligand binding** Finally, TFs up- or downregulate the expression of their downstream targets. TFs have *A* or *X* as their ligand. TFs bound to their ligand exert a regulatory effect *eff*_*bound*_, while ligand-free (apo) TFs have an effect *eff*_*apo*_. These values evolve independently. The fraction *W* of a TF in a particular state depends on the concentration of the ligand and the binding constant *K*_*d*_:

9 $$ W_{{tf}_{bound}} =\frac{[{ligand}] \cdot K_{d}}{1+[{ligand}] \cdot K_{d}}  $$

10 $$ W_{{tf}_{apo}} = 1 - W_{{tf}_{bound}}   $$

**Regulation** The fraction of time (*V*) that an operator is bound by an upstream TF that is either in a particular ligand binding state *σ* depends on the concentration of the TF and its affinity for the operator *K*_*b*_. It is

11 $$ V_{{tf}_{\sigma}} =\frac{W_{{tf}_{\sigma}} \cdot K_{b}}{1 + \sum^{}_{i} \cdot K_{b_{i}}},   $$

where the denominator is a binding polynomial of all TFs (*i*) that bind this operator with affinity $K_{b_{i}}$.

Gene expression can be regulated or unregulated. Regulation depends on TF binding on the operator of a gene. If a TF binds, it can have two different effects, *eff*_*bound*_ and *eff*_*apo*_, depending on ligand binding state of the TF. The regulated effect is the product of its ligand binding state effect (*eff*_*bound*_ or *eff*_*apo*_) and the fraction of time, *V*_*tf*_, that the TF is bound to the operator in either state. The unregulated effect $(1 - \sum ^{}_{tf} V_{tf})$ is 1.. The total regulation is then:

12 $$ {Reg}_{g} =\sum^{}_{tf} \sum^{}_{\sigma} V_{{tf}_{\sigma}} \cdot {eff}_{{tf}_{\sigma}} + \left(1 - \sum^{}_{tf} V_{tf}\right) \cdot 1.   $$

where *tf* is a binding TF and *σ* the ligand binding states of the TF.

#### External resource fluctuation

The external resource concentration fluctuates within the lifetime of the VC. Over a VC’s lifetime, resource concentrations may change up to three times. The probability of changing resource concentrations at least once per generation is 40%. When the external resource concentration changes, the new value is drawn from a logarithmic distribution covering four orders of magnitude.

#### Reproduction

At the end of every generation, the current population is completely replaced by offspring. VCs reproduce with a chance proportional to their fitness, until the population is fully restored. Their fitness is a product of the partial fitness *f*_*i*_ under the three (potentially) different nutrient conditions that they see during their life time:

13 $$ {fitness} = 2^{\prod^{n}_{i} f_{i}} - 1   $$

where *f*_*i*_ is calculated as

14 $$ f_{i}=\frac{1}{ \Delta[A] \Delta[X] }   $$

with *Δ*[*A*] and *Δ*[*X*] the relative distances of the equilibrium concentrations *A* and *X* to their target value, calculated as

15 $$\begin{array}{*{20}l} \Delta A = \frac{\left|\left[A_{eq}\right]-A_{target}\right|} {A_{target}} && \Delta X = \frac{\left|\left[X_{eq}\right]-X_{target}\right|} {X_{target}}.  \end{array} $$

If a cell fails to reach equilibrium within 1000 time steps its fitness is 0.

#### Environmental change

To simulate an environmental change, we change a subset of five parameters: passive diffusion rate, protein degradation rate, catabolic conversion stoichiometry, target resource concentration, and target energy carrier concentration. These can be sorted into two categories: The first category is the homeostasis evaluation set, which contains the target concentration levels for the two metabolites (Eq. 15). Their effect is restricted to the evaluation of the VC fitness. The second category of parameters affects the internal dynamics. These parameters are: the diffusion rate, the degradation rate and the catabolic conversion stoichiometry. Since these parameters affect the molecule concentrations, TFs can sense these changes and alter gene expression within the time step of the environmental change. All novel environments are listed in Table 2.

#### Mutations

VCs are haploid and have a circular genome. After reproduction, offspring undergo mutations with a per gene mutation rate. In this phase, multiple types of mutations can occur. Point mutations target a single attribute of a single gene and affect either the promoter or one of the encoded protein properties. The current value is multiplied with a value randomly drawn from a logarithmic distribution, but will be bounded between 0.1 and 10. Large scale chromosomal mutation target a contiguous stretch of genes with length 1 to a quarter of the genome size. This stretch can be deleted, duplicated or inverted. Whole genome duplications duplicate the entire genome of the individual.

#### Start populations

In previous research, 100 VC populations evolved under fluctuating resource concentrations in the native environment (Table 2). We selected four WT-VCS for continued continued evolution in the current study. Population samples that had been stored during the previously conducted evolutionary runs were selected that had undergone short (1000 generations), medium (4000 generations) and long (11500 generations) term neutral evolution after reaching a high fitness cutoff (>0.85). These served as the starting population seeds for the current study.

### Analysis

#### Constructing the line of descent

Each VC contains a reference to its parent. To construct the line of descent (LOD) that we used for further analysis, we randomly selected an individual in the final populations and traced its ancestors to the first ancestor at the initialization of the experiment. Finally, to avoid the influence of transiently present mutations the last 1000 generations were discarded.

#### Multiple environments analysis

Individuals in the LOD can be subjected to post evolution analysis. To assess fitness we evaluated cells in a standardized set of resource conditions [*A*]_*out*_∈0.1,1.,10.. VC fitness was evaluated both in the environment it experienced during its lifetime and in the alternative environment. Similarly, we tested the effects that mutations would have had in each of the environments and allowed us to identify mutations and genes that can restore fitness in either environment.

#### Functional genome analysis

To reconstruct how the minimal functional network evolves, we reduce the genome (of cells sampled from an ancestor lineage every 100 generations) in an iterative process while retaining the cells fitness. First, TFs with no direct or indirect connection to enzymes or pumps are removed. Next, we assemble a set of genes that can be deleted without fitness costs. These genes are found through single knockout experiments. However, double mutants can have fitness loss even when the two genes that were deleted were neutral to single gene deletion. To isolate only genes that are neutral even with double mutants, we check all possible double mutants combinations for each single knockout gene. If a double mutant is not as fit as the ancestor, both genes are considered functional and removed from the list of potentially removable genes. This process is repeated until no genes remain with the potential to be neutral. This process is repeated for the two environments the cell was exposed to. Only the set of genes that can be removed without fitness loss in both environments is considered non-functional and removed. The remaining genes are called the functional genome.

## Additional file

Additional file 1 Supplementary figures and tables. (1454 KB PDF)

## Abbreviations

GRN: Gene regulatory network
LOD: Line of descent
TF: Transcription factor
VC: Virtual cell
WGD: Whole genome duplication
WT-VCS: Wild-type virtual cell strain

## References

[1] Buescher JM, Liebermeister W, Jules M, Uhr M, Muntel J, Botella E, Hessling B, Kleijn RJ, Chat LL, Lecointe F, Mäder U, Nicolas P, Piersma S, Rügheimer F, Becher D, Bessieres P, Bidnenko E, Denham EL, Dervyn E, Devine KM, Doherty G, Drulhe S, Felicori L, Fogg MJ, Goelzer A, Hansen A, Harwood CR, Hecker M, Hubner S, Hultschig C, Jarmer H, Klipp E, Leduc A, Lewis P, Molina F, Noirot P, Peres S, Pigeonneau N, Pohl S, Rasmussen S, Rinn B, Schaffer M, Schnidder J, Schwikowski B, Dijl JMV, Veiga P, Walsh S, Wilkinson AJ, Stelling J, Aymerich S, Sauer U (2012). Global network reorganization during dynamic adaptations of bacillus subtilis metabolism. Science.

[2] Kriel A, Bittner AN, Kim SH, Liu K, Tehranchi AK, Zou WY, Rendon S, Chen R, Tu BP, Wang JD (2012). Direct regulation of gtp homeostasis by (p)ppgpp: a critical component of viability and stress resistance. Mol Cell.

[3] Giordano M (2013). Homeostasis: an underestimated focal point of ecology and evolution. Plant Sci.

[4] Wegner A, Meiser J, Weindl D, Hiller K (2015). How metabolites modulate metabolic flux. Curr Opin Biotechnol.

[5] Fawcett JA, Maere S, Van de Peer Y (2009). Plants with double genomes might have had a better chance to survive the Cretaceous-tertiary extinction event. Proc Natl Acad Sci U S A.

[6] David LA, Alm EJ (2011). Rapid evolutionary innovation during an Archaean genetic expansion. Nature.

[7] Schobben M, Stebbins A, Ghaderi A, Strauss H, Korn D, Korte C (2015). Flourishing ocean drives the end-Permian marine mass extinction. Proc Natl Acad Sci.

[8] Ferea TL, Botstein D, Brown PO, Rosenzweig RF (1999). Systematic changes in gene expression patterns following adaptive evolution in yeast. Proc Natl Acad Sci U S A.

[9] Oxman E, Alon U, Dekel E (2008). Defined order of evolutionary adaptations: experimental evidence. Evolution.

[10] Cooper TF, Lenski RE (2010). Experimental evolution with E. coli in diverse resource environments. I. Fluctuating environments promote divergence of replicate populations. BMC Evol Biol.

[11] Bailey SF, Kassen R (2012). Spatial structure of ecological opportunity drives adaptation in a bacterium. Am Nat.

[12] Dhar R, Sägesser R, Weikert C, Wagner A (2013). Yeast adapts to a changing stressful environment by evolving cross-protection and anticipatory gene regulation. Mol Biol Evol.

[13] Dragosits M, Mozhayskiy V, Quinones-Soto S, Park J, Tagkopoulos I (2013). Evolutionary potential, cross-stress behavior and the genetic basis of acquired stress resistance in Escherichia coli. Mol Syst Biol.

[14] Bailey SF, Rodrigue N, Kassen R (2015). The effect of selection environment on the probability of parallel evolution. Mol Biol Evol.

[15] Dunham MJ, Badrane H, Ferea T, Adams J, Brown PO, Rosenzweig F, Botstein D (2002). Characteristic genome rearrangements in experimental evolution of Saccharomycescerevisiae. Proc Natl Acad Sci U S A.

[16] Yona AH, Manor YS, Herbst RH, Romano GH, Mitchell A, Kupiec M, Pilpel Y, Dahan O (2012). Chromosomal duplication is a transient evolutionary solution to stress. Proc Natl Acad Sci U S A.

[17] Chang SL, Lai HY, Tung SY, Leu JY (2013). Dynamic Large-scale chromosomal rearrangements fuel rapid adaptation in yeast populations. PLoS Genet.

[18] Crombach A, Hogeweg P (2007). Chromosome rearrangements and the evolution of genome structuring and adaptability. Mol Biol Evol.

[19] Crombach A, Hogeweg P (2008). Evolution of evolvability in gene regulatory networks. PLoS Comput Biol.

[20] Parter M, Kashtan N, Alon U (2008). Facilitated variation: how evolution learns from past environments to generalize to new environments. PLoS Comput Biol.

[21] Neyfakh AA, Baranova NN, Mizrokhi LJ (2006). A system for studying evolution of life-like virtual organisms. Biol Direct.

[22] Cuypers TD, Hogeweg P (2012). Virtual genomes in flux: an interplay of neutrality and adaptability explains genome expansion and streamlining. Genome Biol Evol.

[23] Cuypers TD, Hogeweg P (2014). A synergism between adaptive effects and evolvability drives whole genome duplication to fixation. PLoS Comput Biol.

[24] Draghi J, Wagner GP (2009). The evolutionary dynamics of evolvability in a gene network model. J Evol Biol.

[25] Hughes BS, Cullum AJ, Bennett AF (2007). An experimental evolutionary study on adaptation to temporally fluctuating pH in Escherichia coli. Physiol Biochem Zool.

[26] Wielgoss S, Barrick JE, Tenaillon O, Wiser MJ, Dittmar WJ, Cruveiller S, Chane-Woon-Ming B, Médigue C, Lenski RE, Schneider D (2013). Mutation rate dynamics in a bacterial population reflect tension between adaptation and genetic load. Proc Natl Acad Sci.

[27] Batut B, Knibbe C, Marais G, Daubin V (2014). Reductive genome evolution at both ends of the bacterial population size spectrum. Nat Rev Microbiol.

[28] López-Maury L, Marguerat S, Bähler J (2008). Tuning gene expression to changing environments: from rapid responses to evolutionary adaptation. Nat Rev Genet.

[29] Hottes AK, Freddolino PL, Khare A, Donnell ZN, Liu JC, Tavazoie S (2013). Bacterial adaptation through loss of function. PLoS Genet.

[30] Laan L, Koschwanez JH, Murray AW (2015). Evolutionary adaptation after crippling cell polarization follows reproducible trajectories. eLife.

[31] Ford CB, Funt JM, Abbey D, Issi L, Guiducci C, Martinez DA, Delorey T, yu Li B, White TC, Cuomo C, Rao RP, Berman J, Thompson DA, Regev A (2015). The evolution of drug resistance in clinical isolates of Candida albicans. eLife.

[32] Botero CA, Weissing FJ, Wright J, Rubenstein DR (2015). Evolutionary tipping points in the capacity to adapt to environmental change. Proc Natl Acad Sci.

[33] McCutcheon JP, Moran NA (2012). Extreme genome reduction in symbiotic bacteria. Nat Rev Microbiol.

[34] Richards VP, Palmer SR, Bitar PDP, Qin X, Weinstock GM, Highlander SK, Town CD, Burne RA, Stanhope MJ (2014). Phylogenomics and the dynamic genome evolution of the genus streptococcus. Genome Biol Evol.

[35] Kashtan N, Alon U (2005). Spontaneous evolution of modularity and network motifs. Proc Natl Acad Sci U S A.

[36] Espinosa-Soto C, Wagner A (2010). Specialization can drive the evolution of modularity. PLoS Comput Biol.

[37] Parter M, Kashtan N, Alon U (2007). Environmental variability and modularity of bacterial metabolic networks. BMC Evol Biol.

[38] Tagkopoulos I, Liu YC, Tavazoie S (2008). Predictive behavior within microbial genetic networks. Science (New York).

[39] Mitchell A, Romano GH, Groisman B, Yona A, Dekel E, Kupiec M, Dahan O, Pilpel Y (2009). Adaptive prediction of environmental changes by microorganisms. Nature.

[40] Kashtan N, Noor E, Alon U (2007). Varying environments can speed up evolution. Proc Natl Acad Sci U S A.

[41] Cullum AJ, Bennett AF, Lenski RE (2001). Evolutionary Adaptation to Temperature. Ix. Preadaptation to novel stressful environments of Escherichia Coli adapted to high temperature. Evolution.

[42] Ketola T, Mikonranta L, Zhang J, Saarinen K, Örmälä A-M, Friman VP, Mappes J, Laakso J (2013). Fluctuating temperature leads to evolution of thermal generalism and preadaptation to novel environments. Evolution.

[43] Caspeta L, Nielsen J (2015). Thermotolerant yeast strains adapted by laboratory evolution show trade-off at ancestral temperatures and preadaptation to other stresses. mBio.

[44] Kuwahara H, Soyer OS (2012). Bistability in feedback circuits as a byproduct of evolution of evolvability. Mol Syst Biol.

[45] Tsuda ME, Kawata M (2010). Evolution of gene regulatory networks by fluctuating selection and intrinsic constraints. PLoS Comput Biol.

[46] Cordero OX, Polz MF (2014). Explaining Microbial Genomic Diversity in Light of Evolutionary Ecology. Nat Rev Microbiol.

[47] Tenaillon O, Barrick JE, Ribeck N, Deatherage DE, Blanchard JL, Dasgupta A, Wu G, Wielgoss S, Cruveiller S, M’edigue C, Schneider D, Lenski RE (2016). Tempo and Mode of Genome Evolution in a 50,000-Generation Experiment. Nature.

[48] Jack CV, Cruz C, Hull RM, Keller MA, Ralser M, Houseley J (2015). Regulation of Ribosomal DNA Amplification by the TOR Pathway. Proc Natl Acad Sci.

[49] de Boer FK, Hogeweg P (2010). Eco-Evolutionary Dynamics, Coding Structure and the Information Threshold. BMC Evol Biol.

