# Supplementary material for: Evolution of evolvability and phenotypic plasticity in virtual cells
Source: BMC Evol Biol. 2017 Feb 28;17:60. doi: 10.1186/s12862-017-0918-y (PMC5329926; doi:10.1186/s12862-017-0918-y)
Supplement: Additional file 1 — Supplementary figures and tables. (1454 KB PDF) [file 12862_2017_918_MOESM1_ESM.pdf]

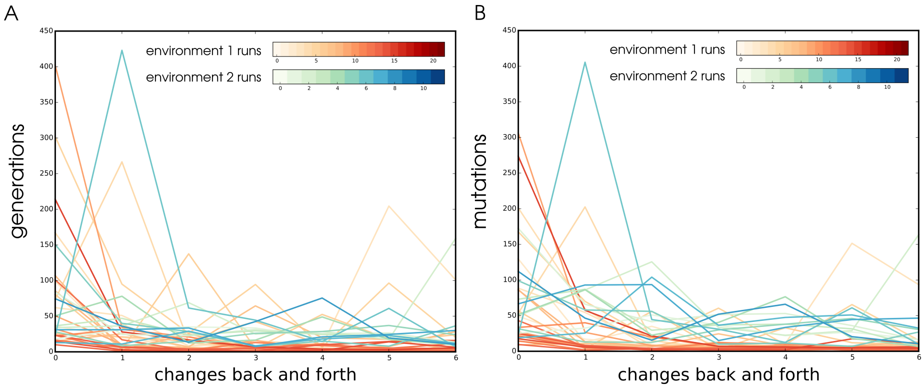

Figure S1: **Generations and mutations needed for readaptation in successfully adapting lineages** Populations under environmental change at 1000 generation intervals between the native and novel environment. As novel conditions, environments 1 and 2 as defined in Table 1 were used. The number of generations (A) and mutations (B) needed to readapt is plotted at every back and forth change of the environment. The values are plotted separately for each WT-VCS.

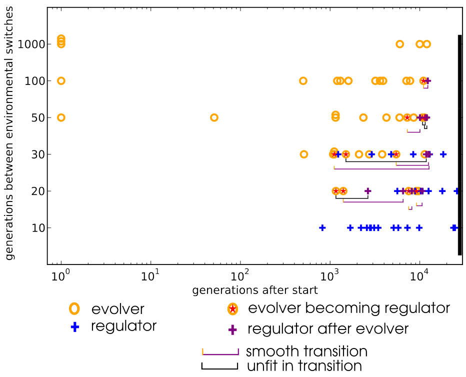

Figure S2: **Time of appearance in generations of adaptive strategies at different ecological time scales** Data are combined from simulations in environments (1,2,3 and 4). The time of appearance in generations of the evolver and regulator strategies in the LOD are plotted for evolutionary simulations under different frequencies of environmental change. The first occurrence of a regulator strategy is recorded if the individuals in the lineage maintain a fitness value > 0.7 in both the native and novel environment. The first occurrence of the evolver strategy is recorded if for 9 out of 10 subsequent changes the fitness first drops below 0.4 and then rises above 0.7 within 10 generations of environmental change. If an evolver strategy arises that subsequently gives rise to the regulator strategy in the same lineage, this event is additionally marked in B with a red star and a line indicating how long and how (purple: no fitness loss, black: temporary failure to reach fitness criterion) the transition to the regulator strategy proceeds.

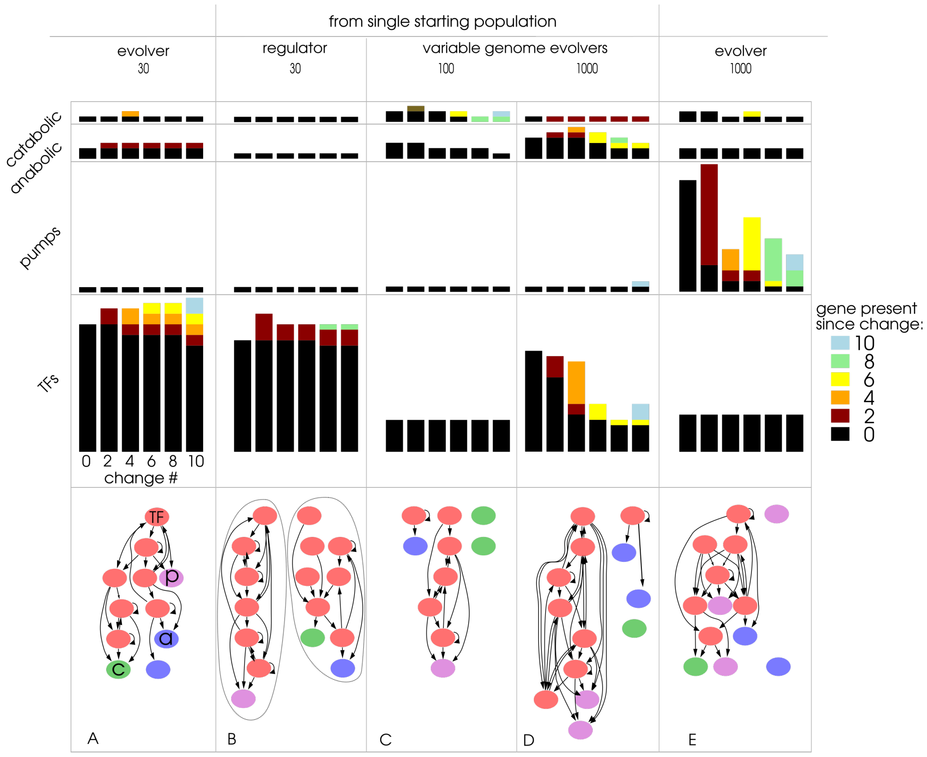

Figure S3: **Evolutionary genome dynamics in environment 1** The top graphs show the conservation of genes in the final ten environmental changes. Bars represent number of genes per gene category and are coloured coded according to time of origin (counted in environmental changes) of genes. Only changes in the last ten changes in the simulation are shown, corresponding to 300, 1000 and 10000 generations, respectively for period 30, 100 and 1000 environmental changes. Bottom graphs show the essential gene regulatory network at the final time point of the simulation. Red: transcription factors, blue: anabolic genes, green: catabolic genes and purple: pumps.

parameter	default	explanation
environmental parameters		
conversion rate	4	rate of conversion A_{in} to X]
passive diffusion	0.1	passive diffusion of A over cell membrane
enzyme-start-concentration	1.0	start concentration of gene products
target-A-in	1.0	target $[A_{in}]$
target-X-in	1.0	target $[X_{in}]$
experimental parameters		
start-A-in	1.0	start $[A_{in}]$
start-X-in	1.0	start $[X_{in}]$
gene-ratios	2.:1.:1.	ratio of TFs:PUMPs:ENZYMEs
assays	3	number of resource concentration fitness checks per generation
enzyme-degradation	1.0	protein degradation rate
dimension	32	population size = $dimension^2$
number-of-genes	10	average genome size of population at initialization
mutation-rate	0.05	per gene mutation rate
change-rate	0.4	resource concentration change rate
switch-delay	0	delay switching the environment, after fitness threshold was reached
size-max	500	genome size above which a penalty will be applied
size-penalty	0.1	penalty per gene on raw fitness data
lifetime	1000	allowed time for the cells to equilibriate their internal state

Table S1: **Parameters used in evolutionary runs to generate WT-VCS** Subsequent continuations use the environmental changes as defined in Table 1 in the main text

Generations between environmental changes	Mean fitness first 30 generations	Pairwise p-values 30	50	100	1000
30	0.69	-	0.038	0.0059	0.0059
50	0.71	-	-	0.0059	0.0059
100	0.77	-	-	-	0.034
1000	0.80	-	-	-	-

Table S2: **Comparison of fitness of evolvers at different frequencies of environmental change** Per 2000 generations the first 30 generations after each environmental switch are averaged, excluding the first and last three. The mean fitness of these is the average of all time points of all populations. We paired the averages of populations at each time point to test the grouped differences with Kruskal- Wallis and used Bonferroni correction to calculate p-values of the differences between populations evolved in the different frequencies of environmental change.

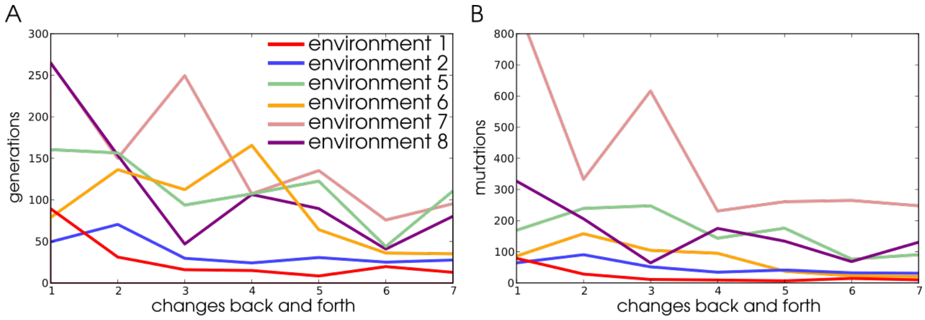

Figure S4: **Generations and mutations needed for readaptation in alternative environments** For environments 1, 2, 5, 6, 7 and 8 defined in Table 1 in the main text, the number of generations (A) and mutations (B) needed to readapt is plotted at every back and forth change of the environment. The values are the median of all simulations in the respective environment, combining all WT-VCS and replicates. Changes between the native and novel environment are at 1000 generation intervals.
